# Gaseous Plastron on Natural and Biomimetic Surfaces for Resisting Marine Biofouling

**DOI:** 10.3390/molecules26092592

**Published:** 2021-04-29

**Authors:** Yujie Cai, Wei Bing, Chen Chen, Zhaowei Chen

**Affiliations:** 1 School of Chemistry and Life Science, Changchun University of Technology, 2055 Yanan Street, Changchun 130012, China; 2201808062@stu.ccut.edu.cn; 2 Advanced Institute of Materials Science, Changchun University of Technology, 2055 Yanan Street, Changchun 130012, China; 3 Institute of Food Safety and Environment Monitoring, College of Chemistry, Fuzhou University, Fuzhou 350108, China; 201310006@fzu.edu.cn

**Keywords:** antifouling, gaseous plastron, super-hydrophobic, biomimetic, antibacterial

## Abstract

In recent years, various biomimetic materials capable of forming gaseous plastron on their surfaces have been fabricated and widely used in various disciplines and fields. In particular, on submerged surfaces, gaseous plastron has been widely studied for antifouling applications due to its ecological and economic advantages. Gaseous plastron can be formed on the surfaces of various natural living things, including plants, insects, and animals. Gaseous plastron has shown inherent anti-biofouling properties, which has inspired the development of novel theories and strategies toward resisting biofouling formation on different surfaces. In this review, we focused on the research progress of gaseous plastron and its antifouling applications.

## 1. Introduction

Marine biofouling is widespread in marine environments and the most serious issue in the shipping industry [1]. Biofouling generally refers to an undesirable colonization of organisms on the surface of underwater structures. It begins within minutes of soaking and goes through several stages, including bacterial adaptation, biofilm formation [2], and the settlement of larger organisms. Fouling organisms refer to the community of microorganisms, plants, and animals attached to the bottoms of ships, buoys, and all industrial installations [3]. Defilade organisms are complex communities composed mainly of stationary organisms, with numerous species, including bacteria, attached diatoms [4], and many large algae, as well as a variety of phyla, from protozoa to vertebrates. Subsequently, more complex macroscopic organisms appear on the immersed surfaces, for example, mussels [5], barnacles [6], tubeworms [7], and so on. There are more than 2,000 species of fouling organisms in the sea environment, the most common of which are algae, hydroids, barnacles, and oysters. Their adhesion has caused serious harm to marine facilities and ships, and greatly restricts the development of the marine economy.

**Figure 1:**
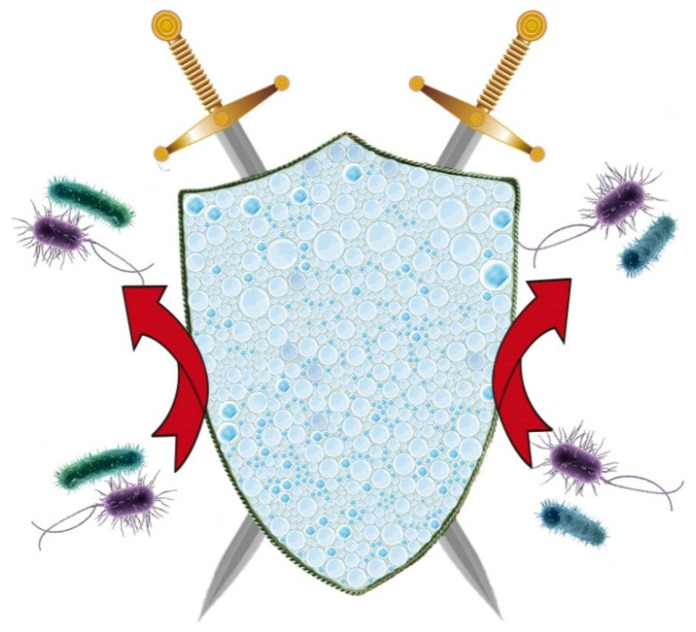
Schematic diagram showing the mechanism responsible for the antifouling of gaseous plastron.

Marine biofouling generally leads to increased sailing resistance and fuel consumption, blocking of ocean transport pipelines, and disabled ocean instruments. Efficient antifouling strategies are critical to maintaining operational performance [8], reducing fuel consumption [9] and cleaning costs [10], and decreasing the transfer risk of intrusive marine species [11]. A series of antifouling technologies have been explored by changing surface roughness, wettability, and electrical properties, or imitating the natural defenses of marine organisms. At present, antifouling strategies mainly focus on non-toxic and environmentally friendly technologies [12,13,14]. Non-toxic antifouling strategies that aim to develop low-adhesion and non-adhesion surfaces are more encouraged, as they exert little environmental risk [12]. As a new non-toxic antifouling strategy, gaseous plastron has achieved certain success with a broad-spectrum and long-lasting antifouling effect (Figure 1). Gaseous plastron, also known as a cushion of air, is a thin film of air between substrate and liquid.

The persistent underwater air layer has evolved in many aquatic and semi-aquatic organisms to adapt to underwater drag reduction or underwater respiration. Five criteria are required for long-term underwater air retention [9], namely, hair-like structures [15,16], hydrophobic chemistry [17,18], undercuts, elasticity of the structure, and heterogeneous hydrophilic tips on superhydrophobic surface [19]. Attachment point theory describes surface interactions based on the relative contact surface area [20,21] of organisms at the water/surface interface, where the size difference determines the strength of the attachment point. In contrast to the fluid/solid interface, the liquid/air interface does not attract the adsorption of marine organisms regardless of their size [22]. Therefore, creating biomimetic surfaces that maintain trapped interfacial air could lead to an alternative class of antifouling coating. The earliest research on gaseous plastron for antifouling dates back to a patent in 1937 [23]. The effective maintenance of gaseous plastron is an important factor that determines its practical application. In this review, we will discuss the factors affecting its stability, and how to obtain stable gaseous plastron by virtue of current advanced technologies. Due to the advantages of broad-spectrum and ecological interest, gaseous plastron has been gradually used in the field of marine antifouling and preventing bacterial adhesion, as shown in Figure 1. In this review, some natural examples of gaseous plastron are discussed in detail to explain how organisms can skillfully use bubbles to survive and adapt to the environment from the macro- to the nano- scale, and how we can learn from such natural processes to design biomimetic artificial antifouling interfaces with similar characteristics via tuning the microstructures and chemical compositions.

## 2. Factors Influencing the Formation and Maintenance of Gaseous Plastron

When a superhydrophobic coating is immersed in water, it can maintain a layer of trapped air to separate the solid from the surrounding liquid. This air layer, also known as gaseous plastron, has shown great potency in continued antifouling, corrosion resistance, and drag reduction. The formation and durability of gaseous plastron is determined by many factors, including the surface properties like wettability and micro/nano-morphologies.

### 2.1. Surface Wettability

**Figure 2:**
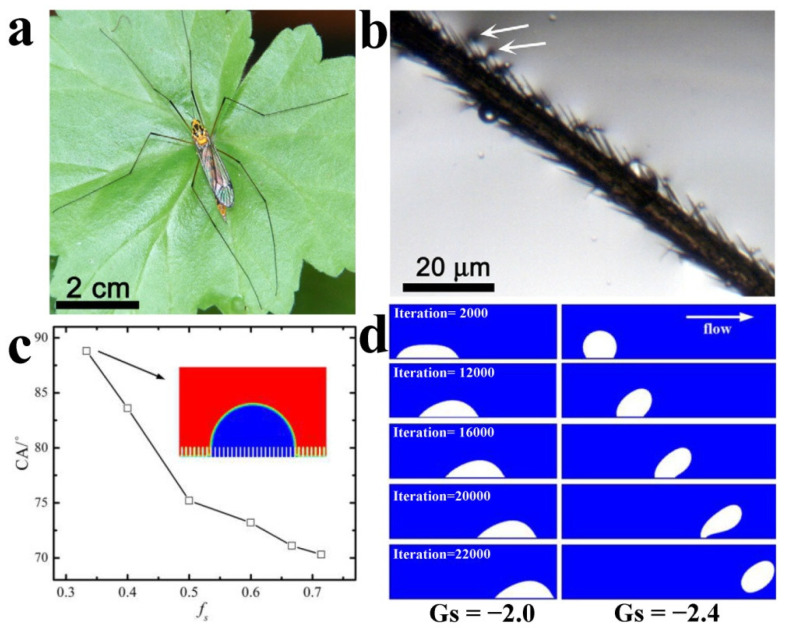
(**a**) With extremely long legs, the cranefly has a large contact area with the leaf. (**b**) The optical image shows a cranefly hair on one side of the leg dimpling the water surface (arrow) [28]. Copyright 2011, *The Journal of Experimental Biology*. (**c**) The relationship between CA and solid area fraction (Gs = −2.1). (**d**) Evolution of bubbles under different solid–liquid interactions. *f_S_*: solid area fraction; Gs: fluid–solid interaction strength. Reproduced with permission [30]. Copyright 2017, *International Journal of Numerical Methods for Heat & Fluid Flow*.

Superhydrophobicity is a surface property that can be realized by a combination of low surface energy and micro- or nano-surface roughness. Superhydrophobicity is reflected by water droplets forming beads on solid surfaces with a contact angle (CA) of more than 150°. When superhydrophobic surfaces are submerged in water, they can trap air within their micro- or nano-structures, creating a gaseous plastron between solid-water interfaces. During the formation of the gaseous plastron, the underwater surface appears to be wetted by bubbles [24]. In fact, on the macroscopic scale, the diffusion of bubbles on a superhydrophobic surface can be regarded as complementary to wetting droplets [25]. In liquid medium, the contact state between the substrate and liquid, namely the solid/liquid/gas three-phase contact state or solid/liquid two phase-contact state, is the key to the behavior of bubbles on the nanoscale substrate [26]. Superhydrophobicity is necessarily and inevitably related to the maintenance of gaseous plastron, such as the lotus leaf and *Salvinia* underwater. Wettability of air bubbles can be seen on the surface of many patterns occurring in nature [27]. For example, the cranefly is a kind of obvious moisture-lover and will perch in a variety of places with water, resting in damp shade, or hanging from leaves on top of water sources, as shown in Figure 2a [28]. The cranefly leg can float on the water with the hairs on one side of the leg, leaving a dent on the surface (Figure 2b). In order to maintain a high degree of hydrophobicity and buoyancy, the air needs to be kept on the convex surface below the liquid body. The legs have a high *f*_SL_ (fraction of the solid–water interface) and are fixed to the solid-liquid-air contact line, requiring high pressure to exhaust the air. The leg hairs of the craneflies are thick and long, and the adhesion is greatly reduced by surface grooves [29]. The air contained in micro-grooves can increase buoyancy and hydrophobicity, and reduce adhesion to water and other unwanted surfaces as well.

The similarity between bubbles and droplets is of great significance for understanding the properties and behaviors of bubbles on a hydrophobic surface. Du et al. designed various surfaces with different solid–liquid interaction areas and studied the effect of microstructure on surface wettability [30]. The solid fraction was defined as *f_S_* = *s*/(*w* + *s*), where *s* was the width of the microstructure, and *w* was the width of the gap between microstructures. During the simulation, they kept the width of the microstructure constant and changed the width of the gap. When *w* is small enough, *f_S_* approaches 0; in other words, the structural surface is analogous to the slippery surface. Figure 2c shows the change of CA with *f_S_* in the solid–liquid interaction when Gs = −2.1 (Gs: fluid–solid interaction strength), indicating that the simulated fluid is in a Wenzel state. The CA of bubbles increases with the decrease of *f_S_* of bubbles. This phenomenon indicates that when the surface is hydrophobic, the presence of a microstructure will increase the CA of bubbles on a solid surface, and the smaller solid area fraction contributes more to the increase of CA. Figure 2d shows the evolution of bubbles. When |Gs| is small, the bubbles diffuse on the wall. In this surface, the bubbles are stable and hard to blow away (Gs = −2.0). When the surface is hydrophilic, the bubbles are seriously deformed under the same flow conditions, and the contact length gradually decreases until the bubbles are separated from the wall (Gs = −2.4). Therefore, surface hydrophobicity is beneficial to maintain the stability of the gas–liquid interface.

### 2.2. Buoyancy

**Figure 3:**
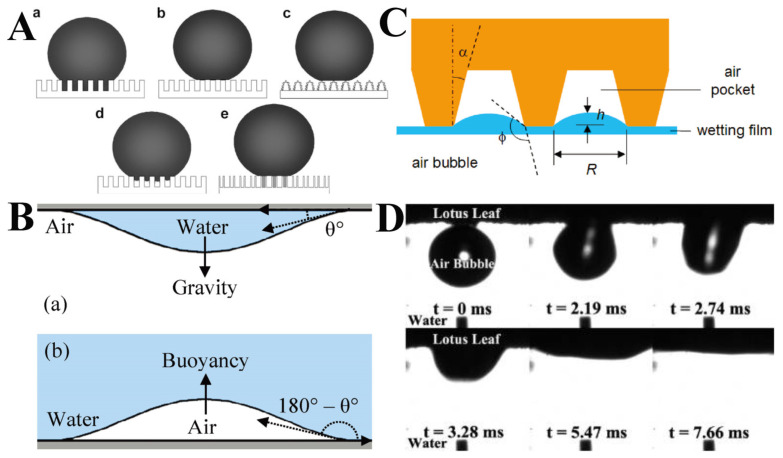
(**A**) Clarification of five superhydrophobic states; (a–e) are Wenzel’s state, Cassie’s state, “lotus” state, a transitional state between Wenzel’s and Cassie’s states, and “Gecko” state, respectively. Reproduced with permission [31]. Copyright 2007, *Advanced Materials*. (**B**) Inverted surface with a contact angle of *θ* and an upright bubble in liquid with a contact angle of 180-*θ*. *θ* is contact angle. (**C**) Schematic of the wetting film between air bubbles and air pockets trapped on a superhydrophobic surface. In this picture, *φ* is the equilibrium contact angle; α is positive opening angle; R is the radius; h is the bath level. Reproduced with permission [39]. Copyright 2011, *Langmuir*. (**D**) A series of optical images showing the rupturing process of a rising air bubble on a lotus leaf surface (t stands for time). Reproduced with permission [37]. Copyright 2009, *Langmuir*.

Wang et al. clarified the definition of a superhydrophobic state in order to better understand the interesting superhydrophobic phenomena on solid surfaces [31]. As for the details of CA hysteresis [32,33,34], superhydrophobic surfaces may have five states, as shown in Figure 3A. On rough surfaces, two superhydrophobic states are common: the Cassie state and the Wenzel state. Surface features at the single microscale or nanoscale usually cause CA hysteresis to some extent, even in the Cassie state [35,36]. In addition, in practice, there is often a transition state between the Cassie and Wenzel states, in which surface droplets will slide when the surface is tilted at a certain angle. Due to its high viscosity, this superhydrophobic state is called the “gecko” state. According to the Young–Laplace relationship, gravity and buoyancy act on the sessile water droplets in the air and the bubbles attached to the inverted surface in the water in similar ways (Figure 3B). For inverted superhydrophobic surfaces, a recent study has shown that air bubbles tend to collapse when buoyancy brings them to the surface [37]. This phenomenon is due to the stability of the wetting film (a thin layer of liquid separating the solid surface from the bubbles) being disrupted. The Laplacian pressure at the three-phase contact line can be approximately calculated by using the equation for capillaries with a height-dependent cross section [38,39]:

(1) $$Pg_{\;}-P_{o}=\rho gH-\frac{2\gamma \cos\left(Ø\;+\alpha \right)}{R+h\;\tan\;\alpha }$$

where *P*_g_ and *P*_o_ are pressure in air pockets and atmospheric, respectively; *ρ* is the density of water; *g* is the acceleration of gravity; *H* is immersed depth; *γ* is liquid–gas interface surface tension; *φ* is the equilibrium contact angle; and *α, R, h* are the geometries as shown in Figure 3C. According to this relationship, the pressure of the captive air pockets will exceed the atmospheric pressure, resulting in cavitation’s coalescence, eventually forming an air bridge and spreading outward, and finally leading to bubbles rupture, as illustrated in Figure 3C. When the length scale of *R* decreases, the positive pressure difference is increased. Wang et al. used high-speed cameras to take a series of optical images of air bubbles [37]. These pictures clearly depict the dynamic diffusion process of air bubbles, as shown in Figure 3D. As the bubbles rise and just touch the surface of lotus leaf (i.e., t = 0), they are almost spherical. However, the contact area between the air bubbles and the lotus leaf surface expands immediately, and the shape of the air bubbles are changed until they completely diffuse to the lotus leaf surface. This spontaneous air rupture is of positive significance for the self-cleaning of superhydrophobic surfaces.

### 2.3. Micro/nano-Morphologies

**Figure 4:**
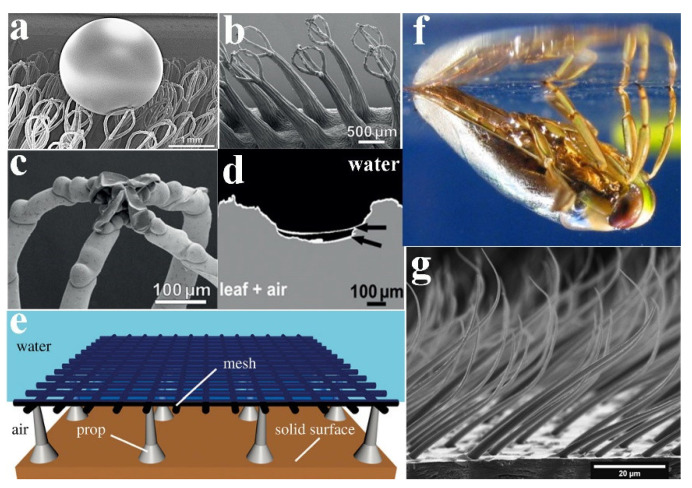
(**a**) SEM of a frozen leaf with a droplet of a water–glycerol solution. (**b**) Eggbeater-shaped structure formed by four multicellular hairs at the terminal. (**c**) The terminal hair is aggregated, forming a hydrophilic patch. (**d**) The air–water interface line (white) formed by *Salvinia* leaf and an air layer in stable fluid. Reproduced with permission [43]. Copyright 2010, *Advanced Materials*. (**e**) Schematic diagram of bionic air-retaining grid surface (AirGrid). Reproduced with permission [44]. Copyright 2019, *Philosophical Transactions A*. (**f**) Lateral observation of the water bug *Notonecta glauca*. (**g**) Setae on the abdominal sternites of *Notonecta glauca*. Reproduced with permission [15]. Copyright 2011, *Beilstein Journal of Nanotechnology*.

Superhydrophobic surfaces can efficiently trap air between the microstructures underwater, forming gaseous plastron [40,41,42]. However, the stability and durability of the gaseous plastron mostly depend on the micro/nano-morphologies of their surfaces. The long-term gas retaining surface (i.e., the *Salvinia* effect) is a hot spot in biomimetic applications. The surface of *Salvinia molesta* (*S. molesta*) is covered with hairs, and the droplets on the *S. molesta* surface are spherical, indicating that the surface is superhydrophobic (Figure 4a). Four hairs gather together to form an eggbeater-like structure. The terminal cells of each hair are collapsed, forming a cap-like structure (Figure 4b,c). At the tip of hairs, hydrophilic patches are formed, and the unique combination of superhydrophobic surfaces and hydrophilic patches can stabilize the air layer and prevent pressure fluctuation. To study the properties of the air–water interface in vivo, Barthlott et al. directly observed the surface top of immersed leaves of *S. molesta* and *Salvinia biloba* in situ by multi-focus optical microscopy [43]. As exhibited in Figure 4d, the hydrophilic patches pinning the air–water interface to the tips of the eggbeater-like structures prevent partial peeling and air loss due to oscillation.

Mail et al. proposed a new constructional principle of studying air-retaining surfaces based on a grid-like structure, which is installed at a certain distance from the surface to seal the air layer [44], as shown in Figure 4e. Depending on the size and geometry of the hydrophobic grid, a layer of air is enclosed between the grid and the surface to prevent water from entering. Ditsche et al. choose *Notonecta glaucaas* as the model organism to study the durability of the air film [15]. Regarding *Notonecta glauca*, in addition to the head, pronotum, and legs, almost all of body has hairy structures. The surface structure of the body is varied, but there are two types of surface protuberances: large, sparse setae and small, dense microtrichia. The base of these setae is in the caudal direction, while the tips are curved in the distal direction (Figure 4f–g). They tested the stability of the air film under hydrodynamic conditions. At the beginning of the experiment, the flow rate was 0.5 m/s, but without the wetting phenomenon. With the increase of the flow rate, the proportion of air cover on the elytra surface decreased slightly (80% at 1.5 m/s). At a high flow rate of 5.0 m/s, 61% of initially covered air remained intact on the surface. The results show that the surface morphologies of the *Notonecta glauca* elytra are suitable to accommodate the air film under hydrodynamic conditions, and keep the film stable under hydrodynamic conditions. To sum up, these natural bio-surfaces provide inspiration for the construction of biomimetic surfaces that could form stable gaseous plastron.

## 3. Antifouling Strategy Leveraged by Natural Organisms Bearing Gaseous Plastron

Over millions of years, optimized superhydrophobic surfaces have evolved in plants and animals, and now serve as models for the development of biomimetic materials. There are a tremendous number of bubbles forming on these natural surfaces, such as lotus leaves, flower petals, fish scales, and so on. Their structures combined with bubbles can take away the biofouling attached to their surfaces or prevent pollutants from sticking to them, ultimately keeping the surface clean. The following cases will help to better understand the mechanisms and processes of gaseous plastron, and how these creatures can be used as biomimetic prototypes of artificial antifouling materials.

### 3.1. Lotus Leaf

**Figure 5:**
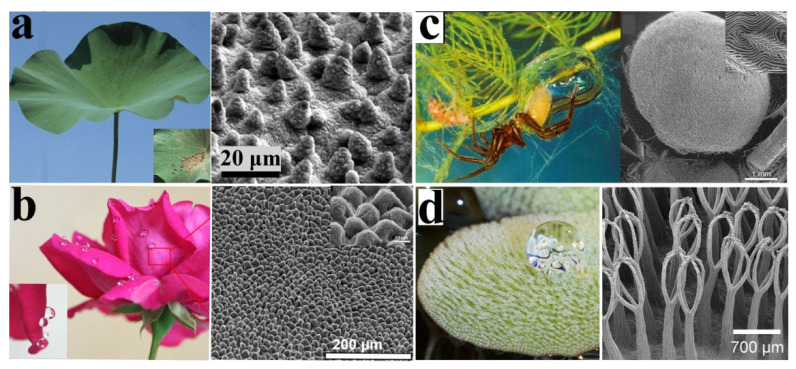
(**a**) Images of a superhydrophobic lotus leaves with self-cleaning properties and scanning electron microscopy (SEM) images of micro-/nano- surface structure. Reproduced with permission [59]. Copyright 2009, *Progress in Materials Science*. (**b**) Real images of red rose petal and water droplets on the petals and SEM images of the surface of a red rose petal showing a periodic array of microstructures. Reproduced with permission [60]. Copyright 2011, *Thin Solid Films*. (**c**) Water spiders have air-sensitive hair on their bellies and legs. Reproduced with permission [61]. Copyright 2018, *Nanoscale*. (**d**) SEM images of *S. molesta*: upper part of densely hairy leaf surface and eggbeaters. Reproduced with permission [62]. Copyright 2014, *Beilstein Journal of Nanotechnology*.

Over the past few decades, the self-cleaning capacity of plants has intrigued researchers. Lotus leaf is an outstanding example of such a characteristic and has attracted great attention [45]. Guo et al. studied the “lotus effect” (Figure 5a) and deduced in their continued research that its self-cleaning property was a result of its microstructures [46]. The multi-layer rough structures on the surface of the lotus leaf led to the residence of an air film between the solid phase of the lotus leaf surface and the liquid phase of the water drop. In the lotus effect, water droplets spontaneously roll down at a tiny angle rather than adhering to these surfaces. The synergistic effect of the multi-scale surface structure and hydrophobicity of the outer epidermal wax makes the droplets have a higher water contact angle and a smaller sliding angle, manifesting as superhydrophobicity and low adhesion. The droplets on the surface are almost spherical and can roll freely in all directions. Meanwhile, they can absorb dust particles and stains, creating a so-called self-cleaning effect.

Jiang et al. first observed the phenomenon of air bubbles bursting on the surface of lotus leaves, known as the “bubble bursting effect” [37]. The micro-papillae surface is immersed in water and keeps a certain distance from the rising air bubbles. Air pockets are captured in the convex part of the lotus leaf surface [47,48,49], which forms a protruding water/air interface over the micro- and nano-structures. As the air bubbles rise close to the leaf surface, the wetting film gradually becomes thinner. The attenuation of the wetting film eventually leads to the coalescence of the air bubbles to capture air pockets among the asperities, leading to the formation of a three-phase contact line (TPCL) [50,51]. Thereafter, the air bubbles will spread along the gas bridge between the air bubbles and the leaf surface [26], and the TPCL is propagated until the air bubbles are fully spread out. This phenomenon is similar to the wetting phenomenon of water droplets in the air. The bubbles quickly diffuse and wet the surface [52]. The diffusion of bubbles on the lotus leaf surface can be regarded as the wetting of water droplets on the leaf surface [53].

### 3.2. Flower Petals

The self-cleaning phenomenon is usually explained by the synergistic effect between the special micro/nano-structures and low surface energy, which is characterized by superhydrophobicity with a high contact angle (>150°) and low slip angle (<5°). In contrast to the popular “lotus effect”, this phenomenon was defined as the “petal effect” (Figure 5b). The petal-effect state (the Wenzel state) means that a droplet is impregnated on a rough substrate, making the rough surface wet. Thus, the air is trapped under the droplet in a Cassie state. Air bubbles can attach to superhydrophobic rose petals through a strong pinning force to maintain the pinning effect, because the surface of the petals is composed of micro papillae and nanofolds with a special layer of rough structure. Ishida et al. found that rose petals have the ability to capture bubbles when they are immersed in water, and quickly stabilize the bubbles on the surface [47]. This phenomenon is known as the stable “pinning effect” of bubbles [54]. A number of studies have shown that the rough micro/nano multi-layered composite structures of rose petals are crucial to the pinning effect property. Microscale rough protuberance and nano-papillae on the surface could form an air cushion layer underwater. When bubbles are close to the petal surface, the air cushion induces air bubbles to coalesce into one, causing the bubbles to be fixed on the petal surface. The macroscopic manifestation of this phenomenon is the CA hysteresis of bubbles, which causes it to adhere to the petal surface. When petals follow the Cassie model, air bubbles will adhere to the petal surface [55,56]. In addition, the size of micron-scale protrusions and the spacing between them are the main factors that determine the pinning effect of bubble stabilization. In addition, the size and spacing of the micro-scale are the main reasons for the stable pinning effect. Therefore, the microstructure mainly affects the superhydrophobicity of rose petals, while the nanostructure is an important factor for the high adhesion of rose petals.

### 3.3. Water Spider

The water spider (*Aqugyroneta aquatica*) is one kind of spider that lives under water almost all of its life [57]. The tough silk produced by water spiders is not used to make a net, but to form a bell-shaped dwelling in the water, called a diving bell. To make the bells stronger and fuller, spiders inject air into them in a special way. Water spiders use the hydrophobic hair on their abdomen and legs to capture air on the surface of water, and then crawl back under the water to inject air into the diving bell (Figure 5c). There are nanometer-sized layers of vertical dendritic structures in the hairs of water spiders, and the regular arrangement of their abdomens is super-aerophilic, which is a key factor determining their ability to easily collect air [58]. When a water spider is immersed in water, a thin layer of air forms on the special micro/nano multi-layer structure of its abdomen. It looks like a layer of net wrapped around the surface, enabling the transport and store of air.

### 3.4. Salvinia

**Figure 6:**
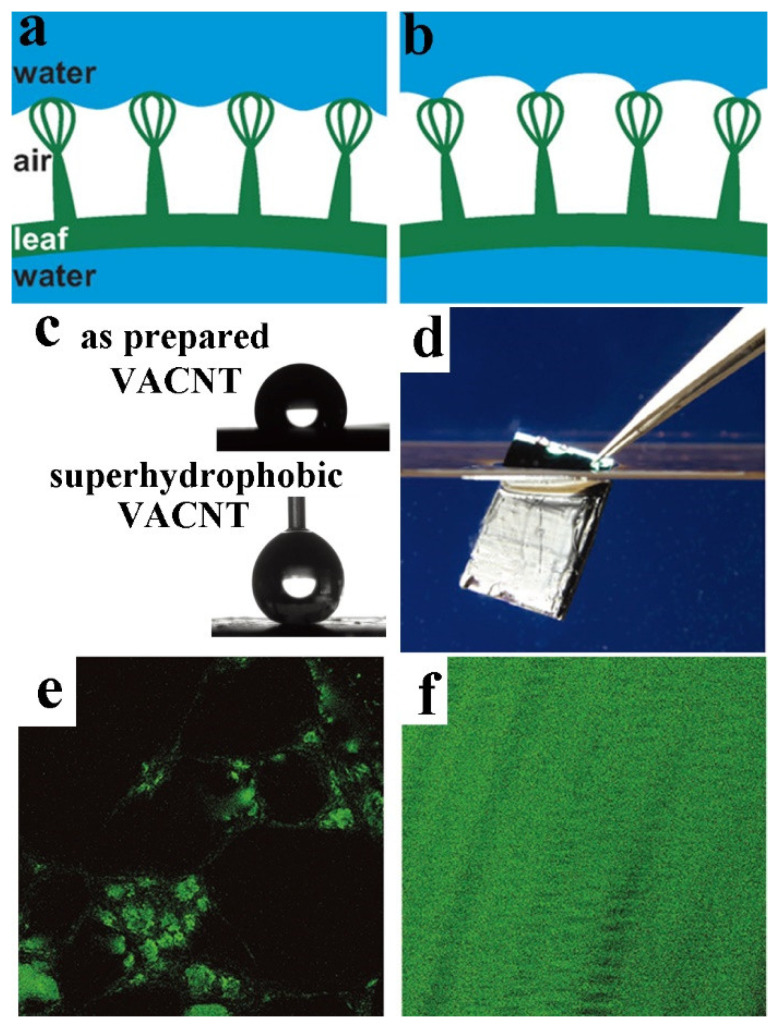
(**a**) Hydrophobic repulsion of hair. (**b**) Hydrophilic tips of the hairs. Reproduced with permission [43]. Copyright 2010, *Beilstein Journal of Advanced Materials*. (**c**) The CA of as-prepared vertically aligned carbon nanotubes (VACNTs) and superhydrophobic VACNTs are 118° and 162°, respectively. (**d**) Superhydrophobic VACNT surface partially submerged in water. (**e**) Confocal microscopy image of the as-prepared VACNT surface and (**f**) the superhydrophobic VACNT surface. Reproduced with permission [68]. Copyright 2017, *Advanced Materials Interfaces*.

In order to adapt to various ecological conditions, such as gas exchange or drag reduction, semi-aquatic plants have evolved the ability to maintain thick (3.5 mm or more) layers of air through complex hierarchies (known as the *Salvinia* effect) [62,63]. The most typical example is the highly complex surface of the floating fern *S. molesta*, which exhibits chemical heterogeneity and immobilizes the air–water interface by superhydrophilic anchoring cells [64]. The floating water fern *Salvinia* has hierarchical architectures dominated by elastic eggbeater-shaped hairs, which has a long-term air retention effect (Figure 5d). These hairs are about 300 to 2200 μm in height, and they are covered with hydrophobic nanoscopic wax crystals appearing as thin rodlets perpendicular to the surface [63,64,65,66]. However, the terminal cells of the hair are not covered with wax crystals, but form hydrophilic tips. This “edge effect” may be responsible for keeping the entire air layer on the leaf. The resulting “*Salvinia* effect”, which is the presentation of hydrophilic tips on a superhydrophobic surface, increases the stability of the air layer at low pressure and prevents the extraction of air bubbles [67]. Figure 6a,b shows a schematic diagram of hydrophobic repulsion (a) and pinning by the attraction of hydrophilic tip of hairs (b) to stabilize the air-water interface.

The *Salvinia* effect means that the air layer remains stable in water [69], which is the result of the complex hierarchical architecture. A similar effect has also been observed for some insects like *Notonecta* [15,16]. Due to the total reflection of light on the air-water boundary, the trapped air layer gives its body a silvery appearance. Babu et al. modified vertically aligned carbon nanotubes (VACNTs) and superhydrophobic VACNTs to a silicon substrate to form a complex (Figure 6c) [68]. They immersed the VACNTs and superhydrophobic-VACNTs-modified silicon substrate in water, and the surface glowed with a silver shine, indicating that a layer of air remained beneath the water. The air layer in the VACNTs’ surface was transient, but it could sustain for several hours in superhydrophobic VACNTs (Figure 6d). Because the bare silicon substrate is flat and hydrophilic, air bubbles do not retain on the silicon substrate, resulting in the absence of any silver sheen. This phenomenon shows the importance of the superhydrophobic property of a surface in maintaining the air layer at the water–air interface. Confocal microscopy measurements of the as-prepared VACNTs and the superhydrophobic VACNTs showed that there were only small air pockets in the VACNTs after they were immersed in water (green area in Figure 6e). In contrast, for the superhydrophobic VACNTs, a flat continuous reflecting air layer was observed, and it expanded throughout the sample area (Figure 6f), confirming the superior air retention capability of the superhydrophobic VACNTs’ structures.

## 4. Application of Gaseous Plastron for Anti-Fouling

Inspired by the above natural phenomenon and summarized principles, a range of new biomimetic anti-fouling technologies have been proposed, including changing surface roughness [70], changing wettability [71], bionic sterilization [72,73,74], and mimicking the natural defenses of marine life [75]. People have been looking for a green way to prevent the attachment of marine organisms. The current non-toxic anti-fouling strategies are mainly driven by interfacial structures and can be divided into two types: foul-release (FR) surfaces and attachment-inhibiting surfaces. The FR surface is designed to reduce the adhesion strength of organisms, and is more effective in dynamic conditions [76,77,78], so it can be applied to fast-moving ships. In contrast, an attachment-inhibiting surface can completely prevent the attachment of marine organisms from the beginning [79].

**Figure 7:**
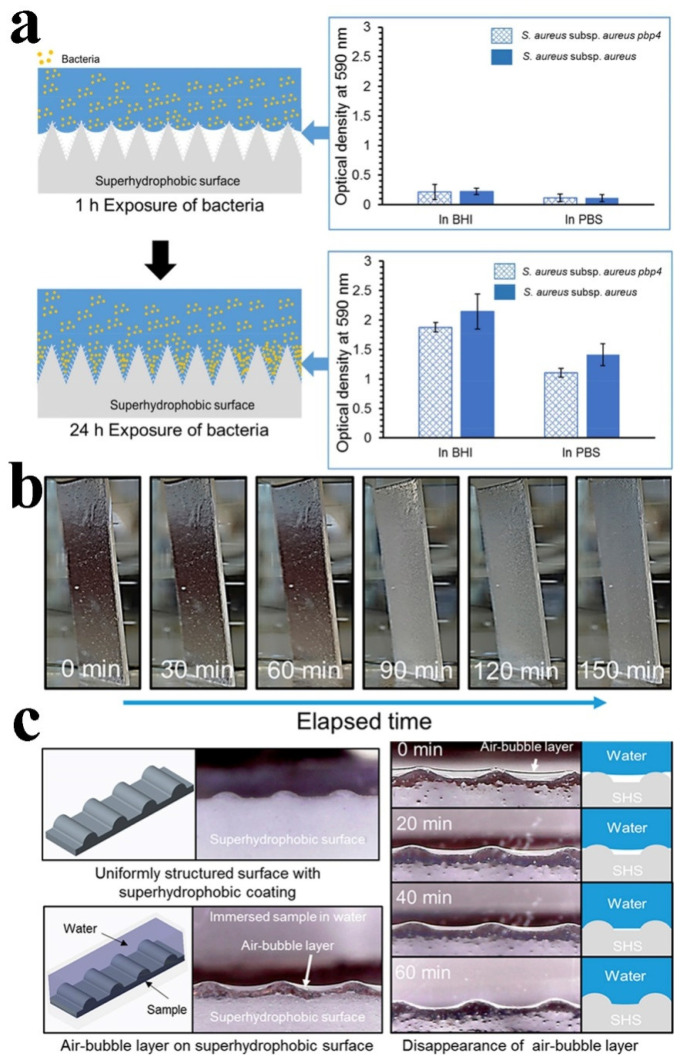
(**a**) The disappearance of the bubble layer before and after the surface adhesion of *S. aureus* and *S. aureus pbp4* (BHI: brain-heart-infusion broth, PBS: phosphate buffer saline). (**b**) The mirror surface is formed through the tortoise-shell effect of a bubble layer. (**c**) Disappearance of bubbles layer between water and surface. Reproduced with permission [80]. Copyright 2018, *ACS Nano*.

Superhydrophobic surfaces have been fabricated in order to reduce biofouling because an entrapped gaseous plastron can reduce the contact between bacteria and a submerged solid surface. Hwang et al. synthesized superhydrophobic materials and tested the adhesion of *S. aureus* on these surfaces, including a wild-type strain and mutant strain (*S. aureus pbp4,* reduced frequency of cross-linked peptidoglycan) [80]. As revealed in Figure 7a, there was no difference in the colonization ability of the two strains at 1 h, but the colonization of mutant *pbp4* was significantly less than that of the wild-type strain after 24 h incubation. This suggests that the bubble layer that initially existed on the superhydrophobic surface inhibited the adhesion and prevented the bacteria from recognizing the surface. However, the gaseous plastron began to disappear after 60 min, and completely disappeared after 150 min (Figure 7b). Figure 7c showed the shape of the bubble layer wrapped around the superhydrophobic surface. The bacteria could not penetrate gaseous plastron, thus preventing bacterial colonization on the surface. Therefore, the long-term stability of gaseous plastron is extremely important in preventing bacterial adhesion.

**Figure 8:**
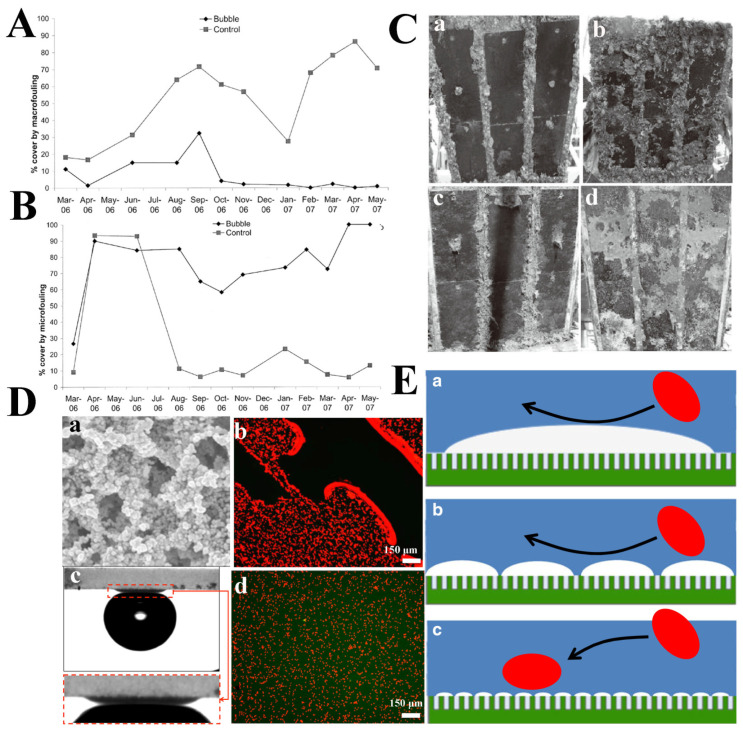
(**A**) Macrofouling cover over time on the air bubbles treatment and the control. (**B**) Microfouling covers over time on the air bubble treatment and the control. (**C**) Comparison of the antifouling effect after 6 or 12 months exposure; (a, c) bubbles treatment for 6 months and 12 months, respectively; (b, d) control experiment for 6 months and 12 months, respectively. Reproduced with permission [81]. Copyright 2009, *Journal of Marine Engineering & Technology*. (**D**) Pores left by thermal decomposition of PMMA emulsion (**a**); images of superhydrophobic surface after incubation with diatoms, 5 h (**b**); contact angle of superhydrophobic surface after ethanol treatment (**c**) and the wetted superhydrophobic surface after incubated with diatoms, 5 h (**d**). (**E**) Schematic diagram illustrating the effect of air pockets’ size on diatom attachment. Reproduced with permission [22]. Copyright 2013, *Biointerphases*.

In liquid, because of the high interfacial tension of water, bacteria are incapable of penetrating the air–liquid interface. This makes it enormously difficult for bacteria to find the location of cell-anchoring sites when there is a large and stable air–liquid interface. The antifouling technology based on gaseous plastron is possible in both laboratory [22] and real marine environments [81]. Scardino et al. used gaseous plastron to protect stationary vessels, and the results showed that air bubble treatment is effective in controlling macrofouling [81]. After 5 months of exposure, there was a noticeable difference in fouling coverage between the group treated with air bubbles (15%) and the control group (64%). During the six months of the experiment, the macrofouling covered on the air-bubbles-treated surfaces was less than 5%. In contrast, the coverage of macrofouling on the surface of the control group increased from 20% to more than 80% (Figure 8A). Compared with macrofouling cover, the area of microfouling covered by the air bubble treatment is larger. In most tests, the coverage of microdirt on the air-bubbles-treated surfaces (mainly consisting slime and hydrate) was greater than 70% (Figure 8B). After 12 months of exposure, the two main fouling organisms that appeared on the air-bubbles-treated surface were erect bryozoans and hydroids (Figure 8C). FR panels exposed to a continuous flow of air bubbles could resist heavy macrofouling for more than 12 months. In contrast, FR panels without air bubbles were heavily polluted and had higher pollution biodiversity. Therefore, using air bubbles to control fouling is a new and environmentally friendly technique, which underscores a broad application prospect.

Superhydrophobic materials can inhibit bacterial adsorption and attachment, because the maintenance of the air layer reduces the effective adhesion area of bacteria. Wu et al. synthesized a superhydrophobic coating and explored the influence of the size and morphology of trapped interfacial gaseous plastron on anti-adhesion of diatoms (Figure 8D) [22]. These surfaces are formed by the accumulation of silica nanoparticles in a hydrophobic gel around polymethylmethacrylate (PMMA) latex particles, which is removed during the curing process, leaving a honeycomb-like structure (Figure 8Da). Florescence microscopy was used to observe the extent of diatoms attached to each superhydrophobic surface. No diatoms were found in the regions of air pockets, while large numbers of diatoms were found in the areas without air pockets (Figure 8Db). To determine the effect of air on diatom deposition behavior, the experiment was repeated on the superhydrophobic surface after treatment with ethanol. The contact angle of ethanol-wetted superhydrophobic substrate is similar to that of a clean glass substrate (Figure 8Dc), and no air pockets remained at the surface. After incubation with diatoms, fluorescence microscopy images indicated that diatoms were evenly distributed on the wetted superhydrophobic surface without aggregations (Figure 8Dd). These results confirm that the bubbles can block the attachment of diatoms. Figure 8E shows the schematic of this effect. Similar to the attachment point theory, the presence of air bubbles reduces the contact points of diatoms on the superhydrophobic surface. By reducing the fluid/surface contact area, the presence of gaseous plastron is a physical barrier against organisms’ adhesion. The gaseous plastron on the liquid/surface interface inhibits the contact of the diatoms to the surface, thus reducing the probability of settlement.

The antifouling performance of gaseous plastron can be further improved by doping a superhydrophobic surface with components. For instance, through the development of soot-coating technology, Esmeryan et al. proposed a novel hybrid superhydrophobic material by embedding silver nanoparticles in the soot matrix [82]. The silver-doped superhydrophobic carbon soot coatings exhibited excellent resistance to microbial adhesion, even after continuous immersion in sea water for three and a half months. This could provide some inspiration for the future development of multifunctional antifouling coating with prolonged potency.

## 5. Conclusions

In natural living systems, air bubbles play a key role in preventing the adhesion of bacteria and algae, thus providing a good solution to biofouling. In nature, the superhydrophobic surface and nano-protruding structures on the surface of lotus leaf, flower petals, and *Salvinia* are beneficial to the formation and maintenance of air bubbles, which confer inherent self-cleaning and antifouling properties. Regarding these natural phenomena, different theoretical models and explanations have been put forward to better understand the mechanisms involved. According to these natural characteristics and principles, researchers have prepared biomimetic antifouling materials, and applied these materials for anti-adhesion and antifouling applications. Antifouling using air bubbles is a simple, non-toxic technology with great potential. Due to the reduction of the fluid/surface contact area, the presence of gaseous plastron is a physical barrier to inhibit microbial adhesion, and the antifouling effect is durable and environmentally friendly. Therefore, the antifouling effect based on gaseous plastron is a bright prospect for engineering applications.

## Data Availability

Not applicable.

## Sample Availability

Samples of the compounds are not available from the authors.
